# Human Amnion-Derived Mesenchymal Stromal Cells in Cirrhotic Patients with Refractory Ascites: A Possible Anti-Inflammatory Therapy for Preventing Spontaneous Bacterial Peritonitis

**DOI:** 10.1007/s12015-020-10104-8

**Published:** 2021-01-03

**Authors:** Mariangela Pampalone, Simona Corrao, Giandomenico Amico, Giampiero Vitale, Rossella Alduino, Pier Giulio Conaldi, Giada Pietrosi

**Affiliations:** 1 Ri.MED Foundation, Palermo, Italy; 2 Department of Laboratory Medicine and Advanced Biotechnologies, IRCCS-ISMETT (Istituto Mediterraneo per i Trapianti e Terapie ad Alta Specializzazione), Palermo, Italy; 3 Section of Histology and Embryology, Department of Biomedicine Neurosciences and Advanced Diagnostics (BiND), University of Palermo, Palermo, Italy; 4 Hepatology Unit, Department for the Treatment and Study of Abdominal Diseases and Abdominal Transplantation, IRCCS-ISMETT, Palermo, Italy

**Keywords:** Cirrhosis, Ascites, Spontaneous bacterial peritonitis, Perinatal tissues, Human amnion-derived mesenchymal stromal cells, Placenta, M2 polarization, Anti-inflammatory therapy

## Abstract

Cirrhosis is associated with dysregulated immune cell activation and immune dysfunction. These conditions modify gut flora, facilitate bacterial translocation, and increase susceptibility to bacterial peritonitis and consequent systemic infections by dramatically affecting long-term patient survival. Human amnion-derived mesenchymal stromal cells (hA-MSCs) exert immunomodulatory potential benefit, and have the ability to modulate their actions, especially in situations requiring immune activation through mechanisms not fully understood. In this study, we aimed to investigate, in vitro, the immunostimulant or immunosuppressive effects of hA-MSCs on cellular components of ascitic fluid obtained from cirrhotic patients with refractory ascites. We found that hA-MSCs viability is not affected by ascitic fluid and, interestingly, hA-MSCs diminished the pro-inflammatory cytokine production, and promoted anti-inflammatory M2 macrophage polarization. Moreover, we found that there was no simultaneous significant decrease in the M1-like component, allowing a continual phagocytosis activity of macrophages and NK cells to restore a physiological condition. These data highlight the plasticity of hA-MSCs’ immunomodulatory capacity, and pave the way to further understanding their role in conditions such as spontaneous bacterial peritonitis.

**Supplementary Information:**

The online version contains supplementary material available at 10.1007/s12015-020-10104-8.

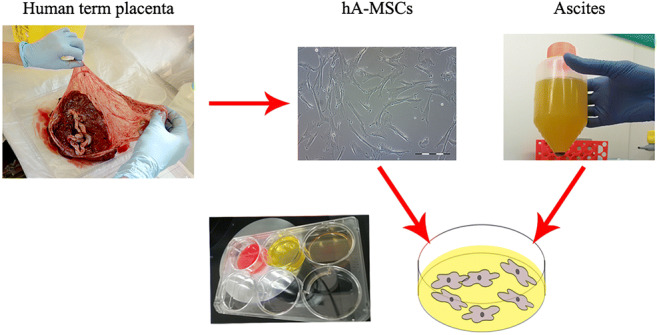

## Introduction

Chronic liver disease (CLD) is currently the 4th cause of death among people aged 45–64 years, and is responsible for 170,000 deaths per year in Europe, and is by far the leading indication for liver transplantation (LT) [1]. CLD patients have over a 50% risk of developing lethal complications within 6 months, and display a considerable reduction in health-related quality of life compared with other chronic diseases [2]. Patients reaching this stage risk complications such as refractory ascites and spontaneous bacterial peritonitis (SBP), which cause about 5–25% mortality while on the LT waiting list [3]. Portal hypertension (PH) is the main complication of CLD, secondary to distorted parenchymal architecture and microvascular dysfunction, which results in increased hepatic vascular resistance and altered blood flow [4] and, in time, determines vasodilation in the splanchnic area, with an increased production of ascitic fluid (AF) within the peritoneal cavity and vasodilating factors, including nitric oxide (NO) [5]. Overproduction of NO is a possible consequence of the altered permeability of intestinal mucosa, which favors the transfer of bacteria and/or endotoxins to the systemic circulation (determining SBP) with activation of the complement system in both plasma and AF, and stimulation of cell-mediated immune responses [6]. The high susceptibility to bacterial infections presented by cirrhotic patients is a frequent complication of liver cirrhosis and increases parallel with its severity [7, 8]. In recent years, the microbiological characteristics of bacterial infections have changed due to an increase of multiresistant germs as a consequence of extended use of primary antibiotic prophylaxis, frequent hospitalizations, and greater use of invasive techniques [9]. The bacteria and their products are able to activate the immune system with increased release of mediators such as NO, TNF-α, IL-6, and IL-1, able to induce the systemic inflammatory response syndrome, progression of which culminates in the multi-organ failure [10]. Cytokines are key components of the immune system, and systemic inflammation is associated with a decreased concentration of anti-inflammatory cytokines in the AF of cirrhotic patients [11]. Moreover, another complication is represented by the so-called “immune paralysis” determined by a reduction of both HLA-DR antigen expression in monocytes and pro-inflammatory cytokine production. Reductions in HLA-DR expression and in vivo production of TNF-α have been described in patients with advanced cirrhosis [12, 13]. It has been shown that monocytes/macrophages obtained from patients with bacterial DNA have an increased expression of IL-6 and TNF-α after LPS stimulation [14].

Regenerative medicine could be helpful in preventing complications related to end-stage liver disease through the application of innovative cell-based strategies. In particular, across recent decades, the use of mesenchymal stromal cells (MSCs) has been considered a new therapy based on their regenerative and immunomodulatory effects [15, 16]. Even if the autologous bone marrow and adipose tissue-derived mesenchymal stromal cells (BM-MSCs and AT-MSCs, respectively) are the most prevalent well-accepted cells in clinical trials [17, 18], the invasiveness of collecting samples in both procedures has led scientists to find other sources of MSCs. For example, extraembryonic tissues, such as placenta [19], amniotic membrane [20–22], and umbilical cord [23–25], which are usually intended as discards of childbirth, have recently been considered new emerging sources. MSCs isolated from human term amnion (hA-MSCs) meet all the release minimal criteria [26, 27] but, compared with MSCs isolated from bone marrow, the placental-derived MSCs have been reported to proliferate faster and more robustly during expansion in in vitro cultures [18]. The hA-MSCs and their derivatives have been demonstrated to be effective in several diseases with underlying inflammatory and fibrotic abnormalities, such as liver fibrosis [28, 29]. Moreover, we recently have shown that the intra-peritoneal infusion of amnion-derived multipotent cells (hA-MSCs and human amnion-derived epithelial cells, hAECs) in rats with advanced liver cirrhosis complicated by portal hypertension and ascites, markedly reduce hepatic inflammation, anti-inflammatory cytokines, oxidative stress and effectively decrease portal pressure and improved liver function tests [30]. Considering all these findings, hA-MSCs could be thought of as a potential cell-based therapy for the treatment of cirrhotic patients.

For this reason, we tested, in vitro, the response of hA-MSCs to AF exposure in order to evaluate their morphology, viability, proliferation, and oxidative stress. Subsequently, further analyses were conducted in order to ascertain the production of both pro- and anti-inflammatory cytokines. In particular, we tested the IFN-γ, IL-12, IL-1, IL-2, IL-4, IL-6, and TNF-α production after hA-MSC exposure at different times with AF compared to the cytokine component present in AF after paracentesis. Moreover, we evaluated whether and how the CD14+ monocytes present in AF may differentiate towards an M1 or M2 macrophage profile during co-culture with hA-MSCs [31] and we further demonstrated that hA-MSCs did favor the shift and maintenance from pro-inflammatory M1 macrophages toward an M2 state in vitro, as expected since M2-like polarization has been proposed in chronic parasitic, viral, or bacterial diseases [32]. The NK and T cells phenotypes were also taken into account.

The obtained results could be the basis to further investigate the therapeutic role of hA-MSCs in bacterial clearance and macrophage phagocytosis as well as their cross talk with immune cells in SBP.

## Materials and Methods

### Patient Ascitic Fluid Collection

AF was obtained from three cirrhotic patients (1 male and 2 females), mean age 64.3 (range 56–72), all in Child B9 class (according to Child-Pugh score) complicated by refractory ascites due to diuretic therapy. Liver cirrhosis etiology was cryptogenic (male), autoimmune and non alcoholic steatohepatitis (females). Patients were admitted every 15–20 days to day-hospital at IRCCS ISMETT (Istituto Mediterraneo per i Trapianti e Terapie ad Alta Specializzazione), in Palermo, Italy to undergo standardized paracentesis and sterile bedside procedure in which a needle is inserted into the peritoneal cavity and AF is removed [33]. An informed consent was obtained from the three patients to participate in the study. AF was collected in the absence of signs of an AF absolute polymorphonuclear leukocyte (PMN) count ≥250 cells/mm3. The AF samples were sent to the microbiology department and resulted negative after 5 days. An amount of AF ranging from 1 to 2 l was collected from each patient, as explained above. A part of the AF was preserved as such, complete in blood cells, and was indicated as A(C). Another part was centrifuged at 500 *xg* for 10 min in sterile 250-ml conical tubes in order to separate blood cells and obtain a supernatant that was indicated as A(SN). The whole blood cell pellets were then stored as a pool. All the samples (A(C), A(SN), and pellets) were stored at −80 °C until use.

### Isolation and Culture of Human Amnion-Derived Mesenchymal Stromal Cells (hA-MSCs)

The hA-MSCs were isolated within 6 h after birth from amnion of human term placentae (*n* = 3) from healthy donors at 36–40 weeks of gestation. Mothers’ informed consent was obtained according to tenets of the Declaration of Helsinki and local ethics regulation (IRRB/58/13, ISMETT Institutional Research Review Board). Isolation was carried out following a well-established protocol [34] with slight modifications. In brief, amniotic membrane was manually separated from the chorion and washed several times in 0.9% sodium chloride (NaCl) containing 1% penicillin/streptomycin (P/S, 100 U/ml / 100 μg/ml) (Sigma-Aldrich) and 2 nM L-glutamine (Sigma-Aldrich). It was then cut into small pieces of 3 × 3 cm that were decontaminated with a brief incubation in 0.9% sodium chloride (NaCl) containing 1% P/S and 2 nM L-glutamine and 2.5% Esojod (Esoform, Italy). The pieces were then washed for 3 min in a solution of phosphate-buffered saline (PBS) (Biowest) containing 500 U/ml penicillin, 500 μg/ml streptomycin, 12.5 mg/ml amphotericin B, 1.87 mg/ml cefamezin (Pfizer, Italy) and 5 min in PBS containing 100 U/ml penicillin and 100 μg/ml streptomycin. Then, decontaminated fragments were incubated for 9 min at 37 °C in Hank’s balanced salt solution (HBSS, Lonza, CH) containing 2.5 U/ml dispase (Corning, NY, USA). The fragments were then incubated for 5 min at room temperature (RT) in Roswell Park Memorial Institute (RPMI) 1640 medium (Sigma-Aldrich) supplemented with 10% heat-inactivated fetal bovine serum (FBS) (Sigma-Aldrich), 1% P/S, 2 nM L-glutamine (complete RPMI 1640). They were subsequently digested with 0.94 mg/ml collagenase A (Roche, Germany) and 20 mg/ml DNase (Roche, Germany) for 2.5 h at 37 °C. The digested tissue was subsequently filtered with both 100- and 70-μm cell strainers (BD Falcon, USA). The sieved cells were pelleted by centrifugation at 150–300 *xg* for 10 min and resuspended in a complete RPMI 1640. After isolation, we evaluated the yield, viability, morphology, and primary cell culture growth. In order to ascertain the purity of the final pellet we analyzed some of the markers commonly used for the detection of MSCs, as previously described in Miceli et al. 2020 [21]. The hA-MSCs were cultured in plastic dishes at approximately 1 × 10^5^ cells/cm^2^; using Chang Medium (Irvine Scientific) supplemented with 1% L-glutamine and 1% P/S at 37 °C and 5% CO_2_. Cell growth was monitored under inverted phase contrast light microscope (Olympus BH-2, Olympus Optical Co., Tokyo, Japan). The attached cells reached confluence 10 days after plating (passage 0). The cells were then trypsinized using a 0.05% trypsin/0.5 mM EDTA solution (Euroclone), and expanded into a T-75 flask for two passages. For our experiments, we used cells at passage 3.

### Exposure of hA-MSCs to AF

The hA-MSCs were cultured for 1 h, 8 h, 24 h, 72 h, and 1 week with both A(SN) and A(C) AF. The reason the cells were exposed to the two different types of ascites samples was to evaluate whether the different conditioned medium, containing the corpuscular part A(C) or depleted of the corpuscular part A(SN), generated changes in the hA-MSC culture. All culturing was carried out in triplicate with three different batches of hA-MSCs and three different ascites samples from cirrhotic patients. The cells grown in complete RPMI 1640 (referred to from herein as “RPMI”) were used as control, while the 1-h time point of exposure was considered as our point “zero” to which we compared all the time-course experiments. All the conditioned media were collected and stored at −80 °C until their use for subsequent analyses. The cells were instead tested by different assays in order to evaluate the proliferation rate, the death events, oxidative stress, morphology, and cytokine secretion. All the subsequent experiments were carried out in triplicate, using three different batches of hA-MSCs and three different AF samples (total experiments replicates, *n* = 9).

#### Cell Morphology and Characterization of hA-MSCs after AF Exposure

In order to evaluate any modification of hA-MSCs after AF exposure, cell morphology and MSC marker expression were noted. When the cells reached 30% confluence, they were cultured with or without ascitic samples or RPMI (as control), and incubated for1 hour, 8 h, 24 h, 72 h, and 1 week. The morphology was followed and assessed under inverted phase contrast light microscope. Digital images were obtained with a digital camera system (Olympus optical imaging, LC-20, Tokyo, Japan). In parallel, the expression of hA-MSC markers was analyzed harvesting the cells from each condition, washing them twice with FACS buffer (PBS containing 0.3% bovine serum albumin and 0.1% sodium azide), and staining them with antibodies against cell surface antigens, such as CD90 and CD73 (see Table 1), for MSC characterization. The cells were then washed twice with FACS buffer, and the analyses were done with FACS Canto II flow cytometer and FACS Diva software version 8.0.1 (BD Biosciences, CA, U.S.A.).

**Table 1:** Monoclonal antibodies used in flow cytometry

Antigen	Conjugation	Clone	Isotype	Manufacturer
CD14	APC Cy7	61D3	Mouse IgG1, k	Invitrogen
CD16	PE	3G8	Mouse IgG1, k	Becton Dickinson Biosciences
CD206	APC	19.2	Mouse IgG1, k	Invitrogen
CD3	FITC	SK7	Mouse IgG1, k	Becton Dickinson Biosciences
CD45	PE CY7	HI30	Mouse Ig1, k	Invitrogen
CD45	PerCP CY5.5	HI30	Mouse IgG1, k	Becton Dickinson Biosciences
CD56	PE	MY31	Mouse IgG1, k	Becton Dickinson Biosciences
CD73	APC	AD2	Mouse IgG1, k	Miltenyi Biotec
CD90	PE	5E10	Mouse BALB/c IgG1, κ	Becton Dickinson Biosciences

#### Apoptosis and Necrosis Detection

In order to analyze cell apoptosis/necrosis after AF exposure, hA-MSCs were cultured with both ascitic samples A(SN), and A(C), and RPMI (as control) for 1 h, 8 h, 24 h, 72 h, and 1 week. After each time point, the cells were detached and Annexin-V-FITC-7-AAD assay kit (Roche Applied Sciences, Germany) was used for staining, following the manufacturer’s staining protocols. Annexin-V fluorescence (indicating the apoptosis) was detected at an excitation wavelength of 488 nm and an emission wavelength of 525 nm, while 7-AAD fluorescence (indicating the necrosis) was detected at an excitation wavelength of 488 nm and an emission wavelength of 647 nm using a BD FACS ARIA II instrument. Analyses were carried out using FACS Canto II flow cytometer and FACS Diva software version 8.0.1 (BD Biosciences, CA, U.S.A.).

#### Proliferation Assay

The proliferation rate was evaluated with CellTiter-Glo Luminescent assay (Promega) and according to the vendor’s instructions. The cells were plated in 96-well Costar Assay Plate black with clear flat bottom (Corning) at a seeding density of 45,000 cells/well using RPMI and incubated overnight at 37 °C and 5% CO_2_. The starting time point was established when the cells were exposed to 100 μl of A(C), A(SN), and RPMI (as control). Each culture condition was carried out and incubated for 1 h, 8 h, 24 h, 72 h, and 1 week at 37 °C and 5% CO_2_. At different time points chosen, 100 μl CellTiter-Glo Reagent was added, and the luminescent signal generated, which was proportional to the amount of ATP present in live cells. The data acquired were analyzed using Spark® Multimode microplate reader (Tecan Trading AG, Switzerland). The resulting data were presented as relative luminescence units (RLU).

#### Intracellular ROS Levels Assay

The oxidative stress was evaluated analyzing the intracellular ROS levels, which were measured by 2, 7′-dichlorofluorescein (2′,7’-DCF-DA) (Sigma-Aldrich) which reacts with intracellular free radicals to generate DCF, a fluorescent product that is retained within the cells [35]. The cells were incubated with both ascitic samples A(SN) and A(C), and RPMI (as control) for 1 h, 8 h, 24 h, 72 h, and 1 week. The hA-MSCs cultured in RPMI for 1 week and then incubated with 200 μM H_2_O_2_ for 60 min were used as positive control of oxidative stress. At different time points, the cells were washed twice with PBS and incubated with 10 mM DCF-DA for 40 min at 37 °C, and then washed again twice with PBS. Fluorescence was detected at an excitation wavelength of 485 nm and an emission wavelength of 530 nm using a FACS Canto II flow cytometer and FACS Diva software version 8.0.1 (BD Biosciences, CA, U.S.A.). The experiments were carried out in triplicate.

### Analysis of Secreted Cytokines

The levels of different paracrine factors involved in inflammation were determined in each conditioned AF after culture of hA-MSCs in A-C at all different time points set and RPMI (as control) using magnetic bead technology from Luminex with the ProcartaPlex™ Human Cytokine Chemokine Growth Factor (Thermo Fisher), according to the manufacturer’s instructions. The data were acquired with xPONENT® 3.1 software for Luminex 100/200 (Luminex Corporation). Concentration of each factor (see the list in Table 2) was calculated by interpolation from standard curves. Results are shown as fold increase of each conditioned medium relative to basal cytokine composition of the three post-paracentesis A-C samples and RPMI (controls). ProcartaPlex™ Analyst 1.0 was used for analyzing the data obtained. For this experiment A-SN was not taken into account, since the absence of immune cells in the ascites would have been a critical issue and not comparable to the reality of post-transplant patient response.

**Table 2:** Panel of cytokines/growth factors detected with Luminex technology

Cytokine/chemokine/growth factor	Conjugation
EGF	PE
IFN-γ	PE
IL-12p40	PE
IL-1β	PE
IL-2	PE
IL-4	PE
IL-6	PE
TNF-α	PE
VEGF-A	PE

### Analyses of Macrophage Polarization, NK and T Cell Proliferation of Cirrhotic Patient-Derived WBCs in Co-Culture with hA-MSCs

Since macrophage polarization towards M2 phenotype is associated with improvement of inflammation and infected state, and is associated with NK and T cell activation, hA-MSCs were co-cultured with white blood cells (WBCs) derived from 3 cirrhotic patients in order to evaluate the effects of hA-MSCs on patient macrophages. The whole blood cells, obtained after centrifugation of the initial ascites and stored at −80 °C until use, were thawed at RT. The red blood cells were lysed through 10 min of incubation with 1X erythrocyte lysis solution (10X Stock Solution: 41.4 g NH_4_Cl, 5 g KHCO_3_, 1 ml EDTA 0.5 M pH 8, in 500 ml double distilled H_2_O) and subsequent centrifugation 300 *xg* for10 minutes. Total WBCs were counted with a hemocytometer. The hA-MSCs (1.53 × 10^5^) were plated in 12-well plates (Corning) using RPMI and incubated at 37 °C and 5% CO_2_ for 2 h, after which, 7.65 × 10^5^ WBCs were plated (hA-MSCs/WBCs ratio 1:5) and co-cultured for 1 h, 8 h, 24 h, 72 h, and 1 week. WBCs only, grown in RPMI at the same time points, were used as control. In order to evaluate macrophage stimulation, the experiments were carried out with and without the addition of 0.1 μg/ml of lipopolysaccharide (LPS).

After the co-culture with hA-MSCs, WBCs were separated from the ascites and harvested, washed twice with FACS buffer, and analyzed by flow cytometry as described above to evaluate the state of macrophage polarization. The cells were stained for the detection of M1 (CD14+ CD16+) and M2 (CD14+ CD206+) profile, NK surface antigens (CD16+ CD56+), and T cells (CD3+).

In parallel, the variation of surface marker expression was evaluated, using the same method, on WBCs present in the ascites alone or stimulated with LPS for the same time points set for the experiment. This allowed us to differentiate the response related to the effect of hA-MSCs only on macrophage polarization.

### Statistics

As described above, all the experiments were done as three independent experiments using hA-MSCs from 3 different donors and 3 different AF (*n* = 9). Data from different groups were compared using an unpaired t-test, while data from the same groups were compared using the 1-h time point as our “time zero,” and applying a paired t-test. For analyzing cytokine production, statistics were applied considering each time points chosen. Statistical software R version 3.6.3 was used for analyzing the data. Results are expressed as the mean ± standard deviation (SD). Differences were considered statistically significant at *p* < 0.05.

## Results

### Morphological and Phenotypic Features of hA-MSCs Do Not Change after Exposure to AF

**Fig. 1:**
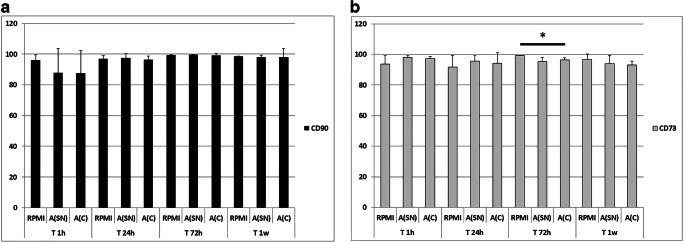
Graph showing that hA-MSCs cultured in standard medium (RPMI) and AF (SN and C) maintained the expression of classical mesenchymal cell surface markers, such as CD90 (**a**) and CD73 (**b**) after 1 h, 24 h, 72 h, and 1 week, with a very slight decrease in CD73 expression after 72 h of A(C) compared with RPMI at the same time point (* *p* < 0.05). Also CD90 decreased after 1 week in RPMI compared with 72 h in the same condition. Values are expressed as means of percentages ± SD

The hA-MSCs cultured in RPMI and in AF were routinely observed under contrast phase light microscope and compared morphologically. It was found that cells in both conditions maintained a fibroblast-like shape. However, as demonstrated in the proliferation assay, the difference between the two culture conditions was also visible. In fact, cells grown in standard culture medium showed a much lower confluence than cells grown in contact with AF at different times (Supplementary Fig. 1). In parallel, it was pivotal to understand whether the cells maintained MSC surface markers. After each time point, phenotype characterization was assayed by flow cytometry (Fig. 1). It was observed that human MSC markers CD90 (Fig. 1a) and CD73 (Fig. 1b) were expressed in all cultures, though a small decrease (2.5%) in CD73 expression was shown after 72 h of A(C) compared with RPMI at the same time point, and in CD90 expression (0.8%) in cells cultured in standard medium (RPMI) after 1 week compared with 72 h (Fig. 1). The other variations were not statistically significant.

### hA-MSC Viability Is Affected after Acute Response to AF Due to Apoptotic Events, but Is Recovered after Long Exposure

**Fig. 2:**
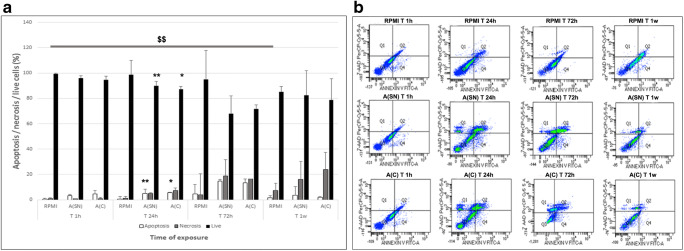
**a** Graph showing the effects of AF apoptotic and necrotic events on hA-MSCs after 1 h, 24 h, 72 h, and 1 week of exposure, compared with standard culture in RPMI. A statistically significant increase of apoptosis (white) was seen after 24 h of exposure to A(SN) (** *p* < 0.01) and A(C) (* p < 0.05), with a concomitant decrease of live cells (black) in both A(SN) and A(C) (** p < 0.01, and * p < 0.05, respectively). However, the resistance to stressing events and decrease of apoptosis are visible as a trend after 1 week, while cells grown in RPMI showed a significant increase of necrosis (grey) ($$ p < 0.01) compared with 1 h. Values were expressed as means of percentages and SD. **b** Representative panel of flow cytometry analyses dot plot showing the possible adaptation of hA-MSCs to AF after long exposure

With the view to using hA-MSCs in the treatment of ascitic patients, it was important to understand not only if AF can affect the viability of the infused cells, but also to integrate the apoptotic, necrotic events after culture in standard medium (RPMI), and AF (A(SN) and A(C)) at the time points chosen (1 h, 24 h, 72 h, and 1 week). We always compared each condition with the RPMI cultures in the same time points, and the different time points to the same condition at 1 h (our” time zero”). As depicted in Fig. 2a, neither apoptosis nor necrosis were significantly changed after 1 h of AF exposure compared with standard condition (RPMI) at the same time point. The response to AF at 24 h, showed a significant increase of apoptosis in hA-MSCs exposed to A(SN) (4.3%, *p* < 0.01), and after exposure to the complete A(C) (4.9%, *p* < 0.05), compared with RPMI at the same time. Moreover, the live cells significantly decreased in both A(SN) (8.9%, p < 0.01) and A(C) (11.7%, p < 0.05). These data seem to contradict previously described findings about a trend of cell viability increase at 24 h. It is likely that the cytosolic ATP increase we obtained using the luciferase method is actually related to apoptotic events that require energy, as described elsewhere [36]. A trend of reduction of apoptosis was observed after 1 week of exposure to both A(SN) and A(C), even if not statistically significant, when compared with the same conditions at 72 h. Moreover, it was observed that when hA-MSCs were cultured in complete RPMI for 1 week, their necrotic event was statistically significantly increased (13.9%, *p* < 0.01) compared with the “time zero” point (1 h), since the medium was never changed during all the experiment. A representative panel of flow cytometry analyses is shown in Fig. 2b, depicting how hA-MSCs were able to respond to AF exposure, reducing apoptosis and necrosis after 1 week of exposure, suggesting a resistance to stress (revealed at 24 and 72 h), also compared with cells grown in RPMI. All these results support the idea of a possible adaptation and survival of hA-MSCs in a complex environment such as AF after long period of exposure.

### Long-Term Exposure to AF Induces hA-MSCs Proliferation Compared with Standard Culture

**Fig. 3:**
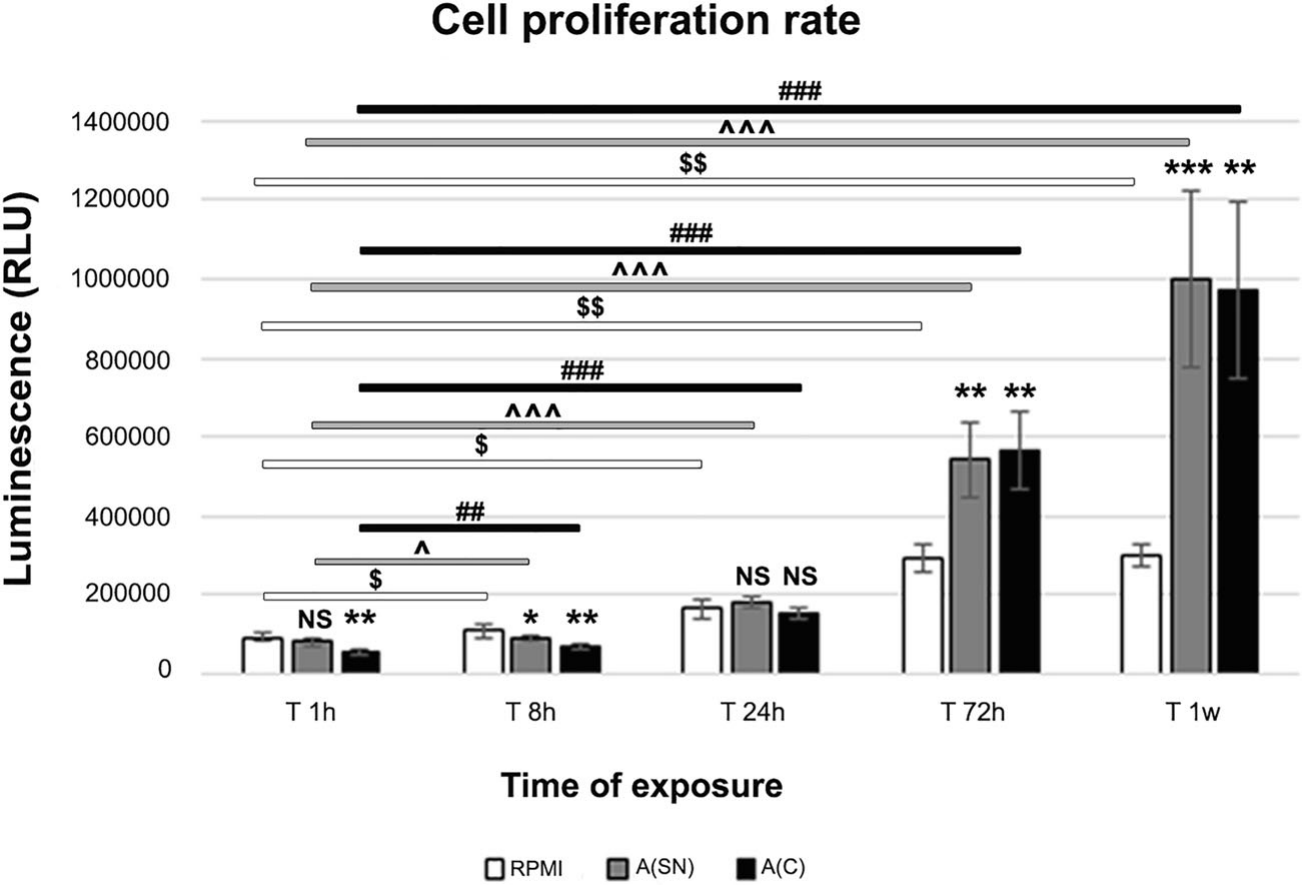
Graph showing proliferation rate of hA-MSCs cultured in complete standard medium (RPMI, white) and in both supernatant, A(SN) in grey, and complete, A(C) in black, ascitic fluid for 1, 8, 24, and 72 h, and 1 week, and expressed as relative luminescence units (RLU). Considering the time point of 1 h as our “time zero”, the cells grown in A(C) were immediately impaired after acute exposure at 1 h (* p < 0.01), while no significance was found when grown in A(SN). After 8 h, the cells cultured in both A(SN) and A(C) reduced their proliferation (* p < 0.05 and ** p < 0.01, respectively). The cells then increased their vitality starting at 24 h, in which no significant differences were seen compared with RPMI in each condition. The positive influence of AF on cell growth was seen after 72 h, with a p < 0.01 (**) for both A(SN) and A(C), and after 1 week, with a *p* < 0.001 (***) for A(SN), and p < 0.01 (**) for A(C). The increase in cell proliferation, considering the same culture condition (RPMI, $, A(SN) ^, and A(C) #) during the different time point sets, was considered statistically significant for p < 0.05 ($, ^, #), p < 0.01 ($$, ^^, ##), and p < 0.001 ($$$, ^^^, ###) compared with the” time zero” starting point of 1 h of exposure. NS: not statistically significant. Values were expressed as means of percentages ± SD

With the aim of using hA-MSCs as cell-based anti-inflammatory therapy for the treatment of cirrhotic patients with refractory ascites, we evaluated the resistance and proliferation rate of hA-MSCs after exposure to AF. As depicted in Fig. 3, the cells showed a statistically significant decrease after only 1 h when exposed to A(C) (around 42% in RLU variation, *p* < 0.01), while at 8 h, both the A(SN) (around 16.5% in RLU variation, *p* < 0.05) and A(C) (around 36.3% in RLU variation p < 0.01) samples underwent a significant reduction of vital cells. However, at 24 h, the effect of AF was normalized, and the differences in cell proliferation were not statistically significant (NS) in any condition. Interestingly, starting at 72 h, AF exposure induced a remarkable increase of hA-MSCs proliferation rate. In fact, at 72 h, a 1.8-fold increase was observed when the cells were grown in both A(SN) (around 85% in RLU variation, *p* < 0.01), and 1.9-fold increase while in culture with A(C) (around 93.4% in RLU variation, *p* < 0.01). After 1 week, the cells tripled their proliferation rate in both A(SN) (around 234% in RLU variation *p* < 0.001) and A(C) (around 224% in RLU variation, p < 0.01). These findings suggest that hA-MSCs are likely initially disturbed by the exposure to AF compared with the standard culture and, then, can recover from the stress, even inducing proliferation. Moreover, the comparison within the same group after each time point highlighted that hA-MSCs were induced, time by time, to proliferate. Considering the time point of 1 h as our reference control, hA-MSCs showed a constant and statistically significant growth in all the media considered. In more detail, cells cultured in RPMI proliferated 1.1-fold after 8 h (*p* < 0.05), 1.7-fold after 24 h (p < 0.05), 3.1-fold after 72 h (p < 0.01), and 3.3-fold after 1 week (p < 0.01). Cells cultured in A(SN) increased their proliferation 1.1-fold after 8 h (p < 0.05), 2.2-fold after 24 h (p < 0.001), 6.6-fold after 72 h (p < 0.001), and 12.2-fold after 1 week (p < 0.001). Cells grown in A(C), were highly influenced by the AF, since they were induced to proliferate 1.2-fold after 8 h (*p* < 0.01), 2.8-fold after 24 h (p < 0.001), 10.5-fold after 72 h (p < 0.001), and 18-fold after 1 week (p < 0.001). The evaluation of VEGF-A showed an increase at 1 week compared with 1 h and 24 h (p < 0.05) (see Table 3), supporting cell proliferation and migration. The effects due to possible cytokines present in the ascites was also evaluated ahead in this study. Since the cells were not negatively affected by AF starting from 24 h, we decided not to consider the 8-h time point in our following experiments.

**Table 3:** Cytokines/growth factors released in complete AF after culturing hA-MSCs at different time points. Values are expressed as means in pg/ml +/− SD

Cytokine/Growth Factor	Time points			
	1 h	24 h	72 h	1w
EGF	1.64	2.07	1.85	1.36
+/-SD	1.76	2.43	1.51	1.34
IFN-γ	0.71	0.77	1.02	1.51
+/-SD	0.38	0.40	0.58	1.24
IL-12p40	0.98	1.45	1.70	1.20
+/-SD	0.20	0.94	1.21	1.32
IL-1β	3.26	3.41	2.15	1.56
+/-SD	0.42	4.79	6.19	2.01
IL-2	0.83	0.89	1.32	1.08
+/-SD	0.39	0.47	0.77	1.27
IL-4	0.79	0.83	1.14	1.12
+/-SD	0.36	0.40	0.47	0.84
IL-6	1.67	3.12	3.21	3.53
+/-SD	0.27	1.81	1.81	6.50
TNF-α	0.81	0.87	1.13	1.44
+/-SD	0.37	0.39	0.61	0.83
VEGF-A	1.30	1.16	2.58	6.80
+/-SD	0.26	1.06	2.86	5.27

### Reduced Oxidative Stress in hA-MSCs as Further Evidence of Positive Adaptation to Long AF Exposure

**Fig. 4:**
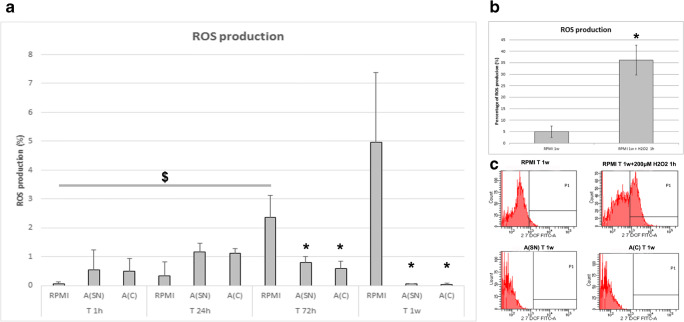
**a** Graph showing the ROS production by hA-MSCs during culture with standard medium (RPMI) and AF (SN and C) for 1 h, 24 h, 72 h, and 1 week. Significant increase was shown only during culture with RPMI after 72 h ($ p < 0.05) and with a trend of continual increase even after 1 week. Interestingly, not only did ROS never significantly increase when cells were grown in both A(SN) and A(C), but their amount significantly decreased after 72 h in both A-SN and A-C (both with p < 0.05, *), and much more also after 1 week in A-SN and A-C (both with p < 0.05, *). Values are expressed as means of percentages and SD. **b** Positive control of ROS production by hA-MSCs at 1 week treated with 200 μM H_2_O_2_ for 1 h and **c** representative plots showing the decrease in ROS production in A-SN and A-C in presence of hA-MSCs after 1 week of culture

Because ascites is characterized by an increase in ROS production, we were interested in the response of hA-MSCs to the ascitic environment and their own production of ROS when cultured in standard medium, A(SN), and A(C) after 1 h, 24 h, 72 h, and 1 week. The graph shown in Fig. 4a illustrates the significant increase in ROS production by cells grown for 72 h in standard culture condition (RPMI) compared with 1 h (2.33%, p < 0.01), and a trend of exceeding ROS production after 1 week of standard culture, likely due to continuous culture without any change of medium. More interesting, not only was there no evidence of ROS production increase when cells were grown in AF, but the exposure to AF at the same time points (72 h and 1 week), revealed a strong adaptation of hA-MSCs, since the amount of ROS was significantly reduced in both A(SN) and A(C) after 72 h (1.6% and 1.82% respectively, *p* < 0.05 for both conditions) and 1 week (4.93% and 4.97% respectively, *p* < 0.05 for both conditions), compared with RPMI at the same time point. A parallel analysis of ROS production was conducted as positive control at a 1-week time point after exposure to 200 μM H_2_O_2_ for 1 h (Fig. 4b), compared with cells not exposed to peroxide, for assuring that cells were significantly (variation of around 31.2%, *p* < 0.05) responsive to oxidative stress. Fig. 4c shows a representative panel of flow cytometry results showing that ROS production was not induced by AF (both A(SN) and A(C)) after 1 week of exposure.

### The Cytokine Composition of AF from Cirrhotic Patients after Long Exposure to hA-MSCs Reflects an Environment Promoting Phagocytosis

**Fig. 5:**
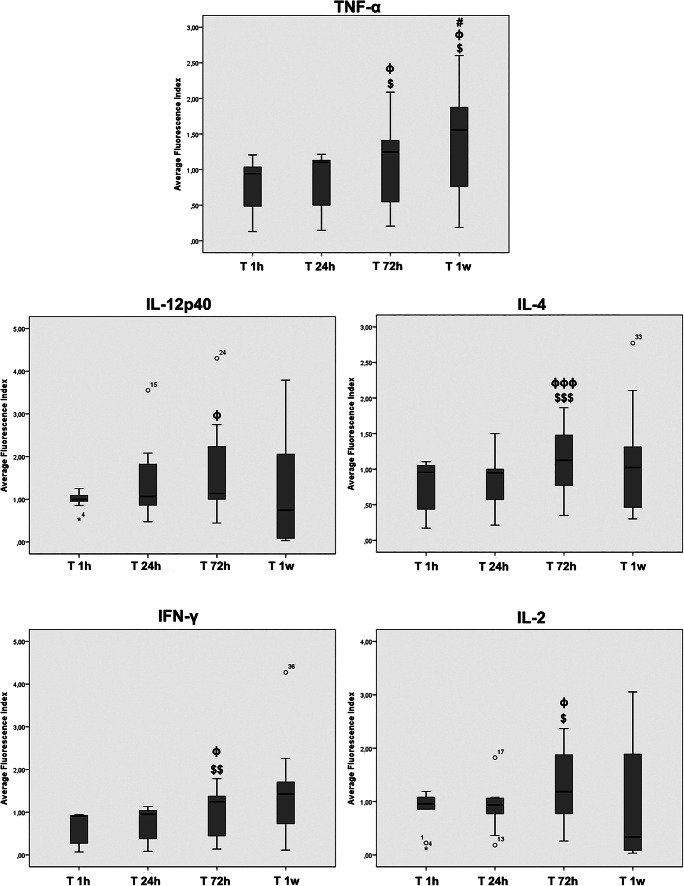
Box plots showing how the inflammatory cytokine release was influenced in ascites after 1 h, 24 h, 72 h, and 1 week in the presence of hA-MSCs. Acute exposure (T 24 h) did not reflect any significant difference compared with 1 h. Significant increases were seen for TNF-α, IL-12p40, IL-4, IFN-γ, and IL-2 after 72 h. Only TNF-α showed a statistically significant increase after 1 week. Statistics were obtained comparing with 1 h ($), 24 h (ϕ), and 72 h (#).Values were statistically significant at p < 0.05 ($, ϕ, #), p < 0.01 ($$, ϕϕ, ##), and p < 0.001 ($$$, ϕϕϕ, ###). Values are expressed as means of fluorescence index± SD

Since AF is characterized by an inflammatory state related to secretion of factors by immune cells already present in ascites, we evaluated the effects of hA-MSCs on cytokine production into complete AF, A(C). In Table 3, the results of all the cytokines considered are listed, while those with statistical significance are also represented in Fig. 5. As depicted, TNF-α, IL-12p40, IL-4, IFN-γ, and IL-2 were statistically significantly changed among the cytokines tested after co-culture with hA-MSCs at the time points chosen. More specifically, the impact on cytokine production was seen after 72 h of co-culture, where we saw an increase of TNF-α compared with 1 h (p < 0.05) and 24 h (p < 0.05), IL-12p40 compared with 24 h (p < 0.05), IL-4 compared with 1 h (*p* < 0.001) and 24 h (p < 0.001), IFN-γ compared with 1 h (*p* < 0.01) and 24 h (p < 0.05), IL-2 compared with 1 h (p < 0.05) and 24 h (p < 0.05). After 1 week no significant modification was noted, except for TNF-α, which exhibited a significant increase when compared with all the preceding time points (all with *p* < 0.05). These findings indicate that interaction with hA-MSCs is able to induce a release of cytokines that are involved in pro-inflammatory environment (TNF-α), also suggesting a possible stimulation of M2-like state of macrophages increase (IL-12p40 and IL-4), and cytotoxic function exerted by NK and T cells (IFN-γ, and IL-2). These effects were reached after long exposure.

### Lymphocyte Induction of M2-like Macrophages, and Proliferation of NK and T Cells Phenotype May Reflect an hA-MSC-Dependent Phagocytic Response in AF of Cirrhotic Patients

**Fig. 6:**
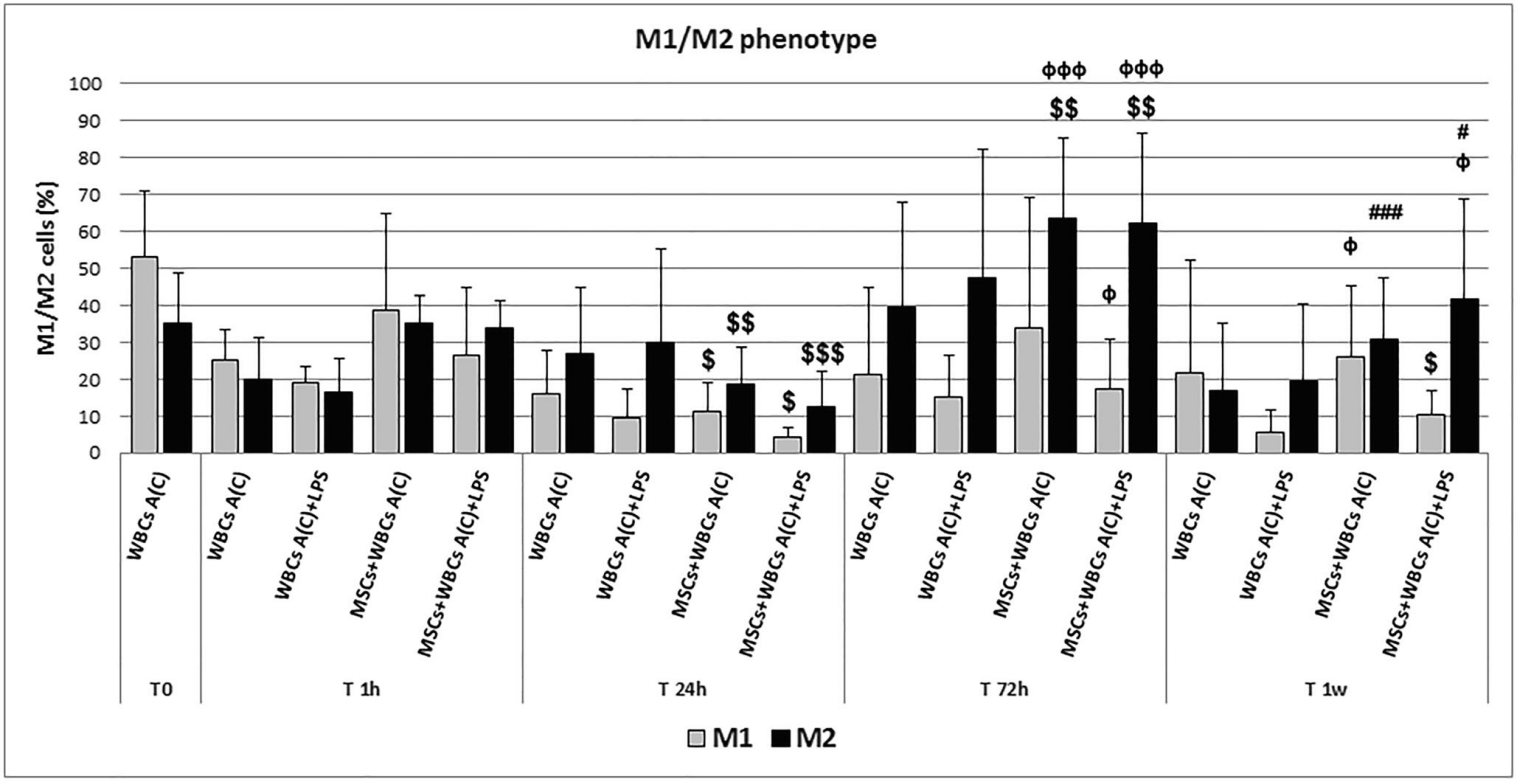
Graph of M1 (grey) and M2 (black) phenotype expressed by WBCs derived from the ascites of cirrhotic patients after paracentesis, A(C) T0, and after co-culture with hA-MSCs for the time point chosen: 1 h, 24 h, 72 h, and 1 week. After 24 h of co-culture, a significant decrease of both M1- and M2-like cells was observed, while a boosted reaction was obtained after 72 h of co-culture. After 1 week, the macrophage composition was restored, and was comparable to 1 h. Statistics were obtained comparing with 1 h ($), 24 h (ϕ), and 72 h (#).Values were statistically significant when p < 0.05 ($, ϕ, #), p < 0.01 ($$, ϕϕ, ##), and p < 0.001 ($$$, ϕϕϕ, ###), and expressed as means of percentages and SD

**Fig. 7:**
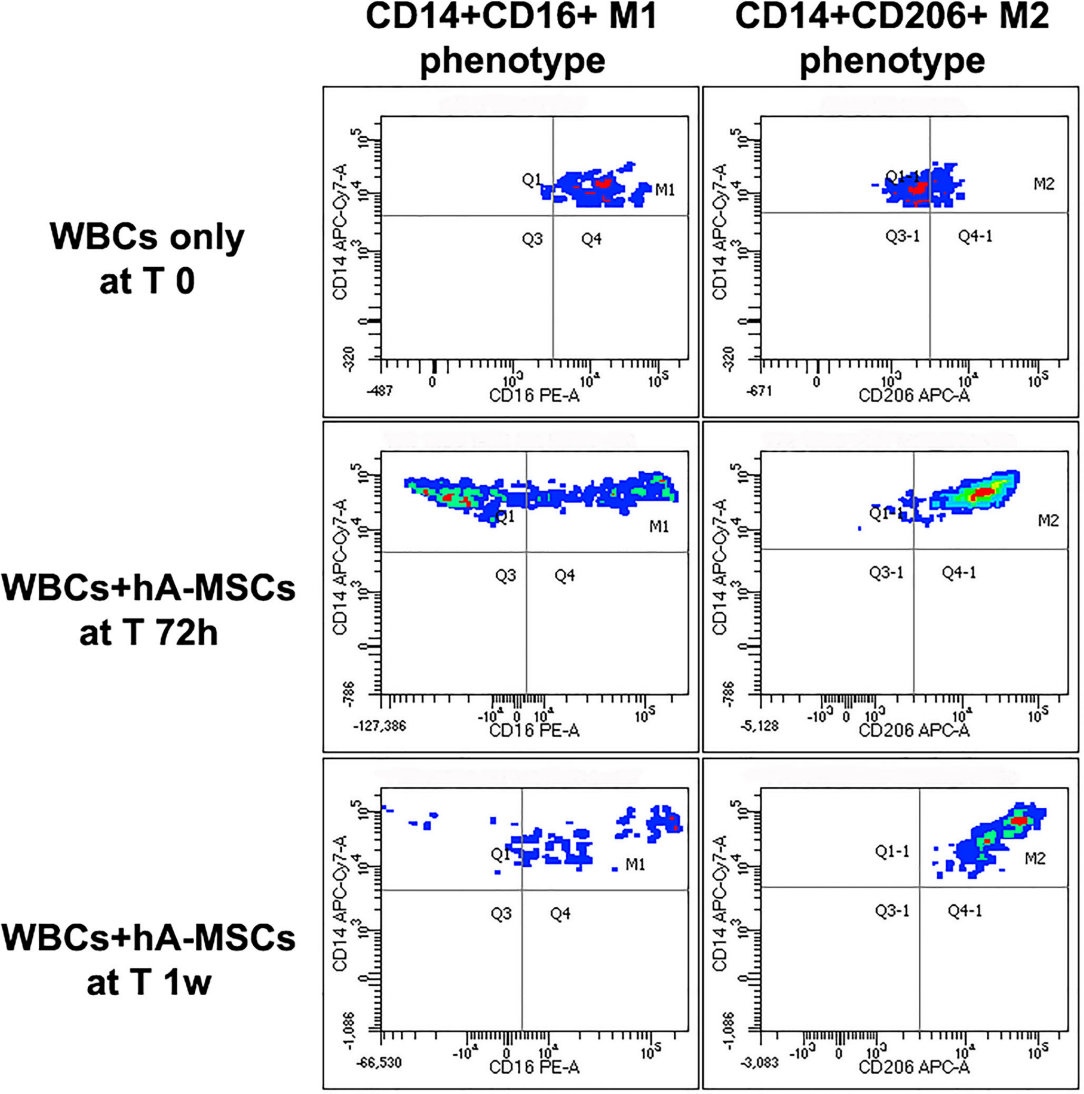
A representative dot plot showing CD14 + CD16+ M1- and M2-like macrophages from total WBCs in post-paracentesis (T 0) AF, in which M1-like cells are prevalent compared with M2-like cells after 72 h of co-culture with hA-MSCs, when the high increase of M2 macrophages was observed, and after 1 week of co-culture with hA-MSCs, in which M2-like cells increased more than M1, even though the latter were still present

The release of inflammatory molecules involved in immune cell response triggered by hA-MSCs was also tested by analyzing the possible boost of those cells involved in phagocytosis. The WBCs derived from the ascites after paracentesis were analyzed by flow cytometry to determine the state of macrophage polarization before and after direct co-culture with hA-MSCs for 1 h, 24 h, 72 h, and 1 week. The LPS was added in parallel conditions in order to create an in vitro SBP model. As represented in Fig. 6, the prevalence CD14+ CD16+ M1-like with respect to CD14+ CD206+ M2-like macrophages in post-paracentesis ascites (T 0), constantly changed during co-culture, using the 1-h time point as reference. In fact, the acute response (after 24 h) revealed that hA-MSCs are able to significantly reduce both M1 and M2 when compared with 1 h, and this response was similar even without LPS (27.6% M1 reduction, with *p* < 0.05; 16.6% M2 reduction with *p* < 0.01) or with LPS (22% M1 reduction with p < 0.05; 21% M2 reduction with *p* < 0.001). Conversely, after 72 h of co-culture, a drastic increase of M2-like macrophages was seen compared with 1 h both in the absence (28.3%, p < 0.01) or presence (28.4%, p < 0.01) of LPS, and with 24 h both in the absence (44.9%, p < 0.001) or presence (49.4%, p < 0.001) of LPS. The M1-like cells increased only in the presence of LPS compared with 24 h only (29.3%, p < 0.05), while no other significant differences were observed. After 1 week, the co-culture with hA-MSCs restored the M2-like phenotype, decreasing their population compared with 72 h both without LPS (32.7%, p < 0.001) and with LPS (20.4%, p < 0.05), and the presence of LPS maintained higher levels of M2-like macrophages compared with 24 h (29%, p < 0.05). With regard to M1-like macrophages, the co-culture with hA-MSCs until 1 week were increased compared with 24 h in the absence of LPS (14.8%, p < 0.05), but with lower percentage in the presence of LPS and compared with 1 h (15.8%, *p* < 0.05). No significant changes were seen in all set time points without the co-culture with hA-MSCs. All these findings suggest a pivotal role of hA-MSCs in the initial and acute impairment of macrophage activation, which was boosted after 72 h and restored with percentages comparable to 1 h, allowing us to postulate that phagocytic events are accomplished between 3 days and 1 week, with a prevalence of M2-related events with respect to M1-related ones, though even the latter were never lost, even after 1 week of co-culture with hA-MSCs, as shown by representative dot plots in Fig. 7.

**Fig. 8:**
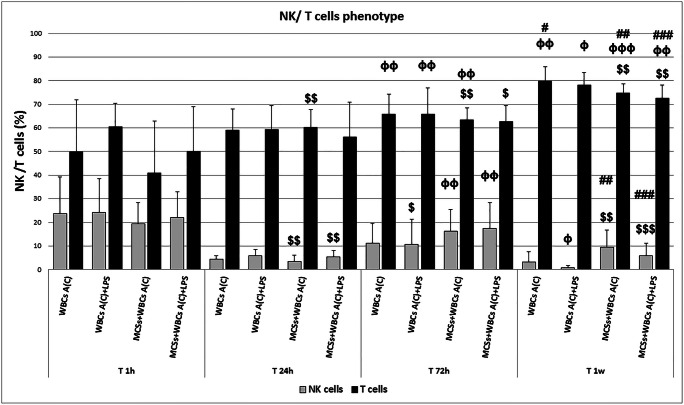
Graph of NK (grey) and T cells (black) phenotypes expressed by WBCs derived from the ascites of cirrhotic patients after co-culture with hA-MSCs for the time point chosen: 1 h, 24 h, 72 h, and 1 week. The co-culture with hA-MSCs significantly determined a decrease of NK and increase of T cells after long exposure (72 h and 1 week). Statistics were obtained compared with 1 h ($), 24 h (ϕ), and 72 h (#). Values were statistically significant when p < 0.05 ($, ϕ, #), p < 0.01 ($$, ϕϕ, ##), and p < 0.001 ($$$, ϕϕϕ, ###), and expressed as means of percentages and SD

**Fig. 9:**
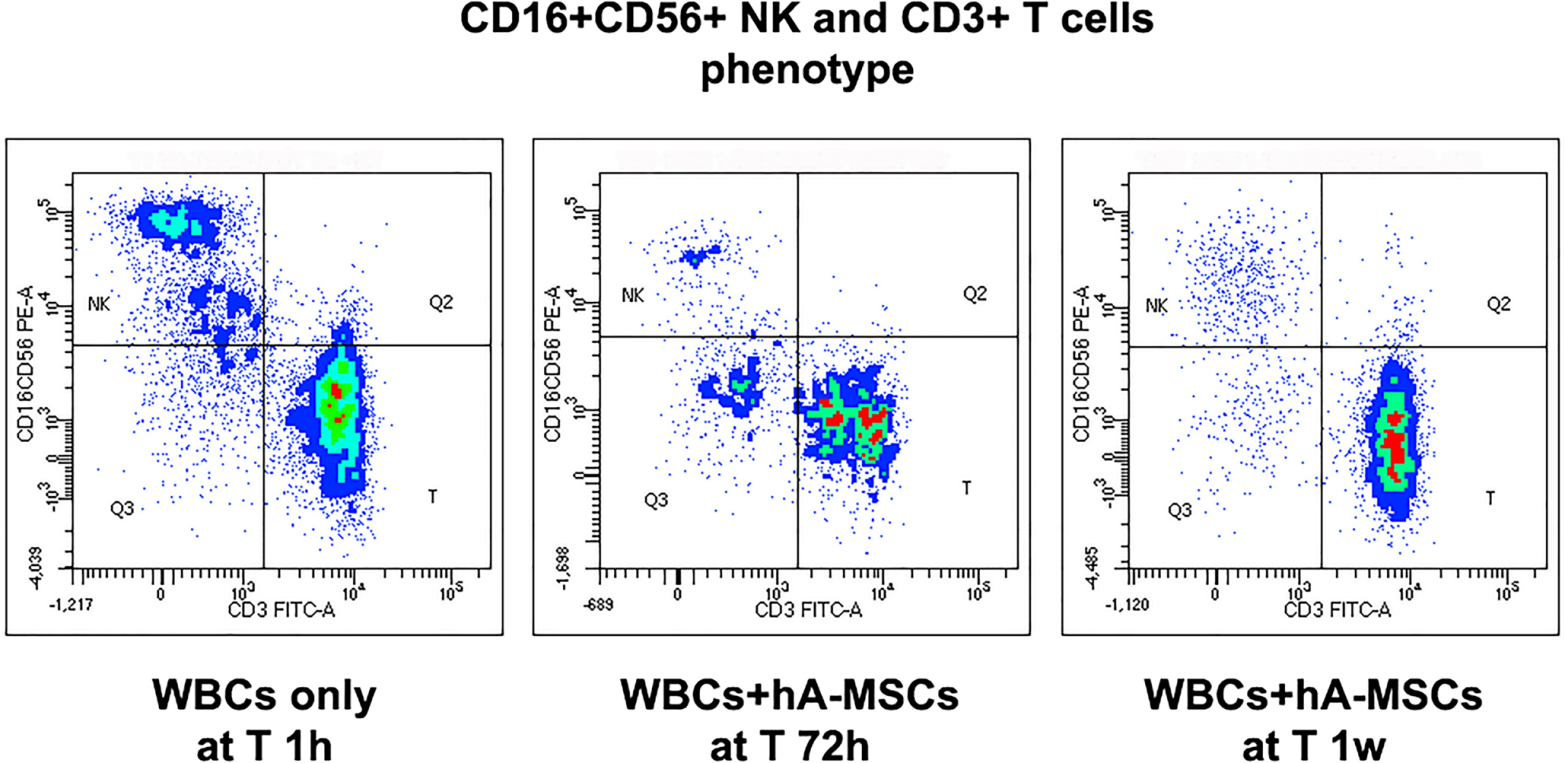
Representative dot plots showing CD16 + CD56 + NK and CD3 + T cells in WBCs after only 1 h of experiment (T 1 h), in which T cells and NK co-exist with a prevalence of T cells. After 72 h of co-culture with hA-MSCs and after 1 week of co-culture with hA-MSCs in which T cells are highly increased compared with NK

Taking into account the cytokines involved in NK and T cell activation, as previously reported, in the same experiments the CD16+ CD56+ NK and CD3+ T cells were also counted at each time point chosen. The results were reported as depicted in Fig. 8. Compared with 1 h, NK drastically diminished, with a concomitant increase of T cells at all subsequent time points. More specifically, after 24 h of direct co-culture of patients’ WBCs with-hA-MSCs, NK were reduced both without LPS (16.1%, *p* < 0.01) and with LPS (16.8%, p < 0.01), while T cells significantly increased in the absence of LPS (19.5%, p < 0.01). After 72 h, during the higher cytokine release and response exerted by M2 macrophages, NK cells still showed lower trend percentages than those at 1 h but, when in co-culture with hA-MSCs, a significant increase of NK was observed compared with 24 h, even without LPS (12.7%, p < 0.01) and with LPS (12.2%, p < 0.01). Conversely, T cells co-cultured with hA-MSCs were higher than after 1 h of co-culture, both without LPS (22.7%, p < 0.01) and with LPS (12.6%, p < 0.05). Compared with 24 h, T cells were statistically higher in all conditions (p < 0.01) except for WBCs co-cultured with hA-MSCs and in presence of LPS. The NK cells decreased significantly in the presence of hA-MSCs after 1 week compared with both 1 h and 72 h in the presence (16.2% and 11.5% respectively, p < 0.01) or absence (10.1% and 6.7% respectively, *p* < 0.001) of LPS. T cells remained permanently higher than other time points, especially when in co-culture with hA-MSCs. In fact, even in the presence of absence of LPS, the increase was observed compared with 1 h (p < 0.01 both without LPS, 34%, and with LPS, 22.7%), 24 h (p < 0.001 without LPS, 14.5%; p < 0.01 with LPS, 16.4%), and 72 h (p < 0.01 without LPS, 11.3%; p < 0.001 with LPS, 10.1%), supporting the results related to the increased release of TNF-α after 72 h and 1 week. In Fig. 9, a representative dot plot panel shows the change in NK and T cell distribution before experiments (post-paracentesis), and after 72 h and 1 week in co-culture with hA-MSCs, but without LPS.

## Discussion

Ascites is one of the major complications of liver cirrhosis in 60% of patients within 10 years from diagnosis [37]. Principal factors contributing to the development of ascites are portal hypertension, hypoalbuminemia, and splanchnic arterial vasodilation [4, 38]. Fibrotic changes and increase of intrahepatic resistance lead to portal hypertension, while hypoalbuminemia related to a reduction of hepatic protein synthesis determines a decrease in intravascular oncotic pressure, thus favoring production of ascites [39–42]. All patients with ascites are at risk of developing SBP, the bacterial infection of AF, with a prevalence of about 20% [43]. The causes of SBP include pathological bacterial translocation from the gut to the systemic circulation due to increased intestinal permeability [44] intestinal bacterial overgrowth [45], change in the quality of bacteria, and the impairment of the local and systemic immune system [46], characterized by reduced activity of mononuclear phagocytes and deficiency of complement components [47, 48]. The immune response therefore determines the release of pro-inflammatory cytokines, which further activate the production of vasodilators by increasing splanchnic vasodilation and promoting, over time, multi-organ dysfunction [49, 50]. The diagnosis of SBP is established by a positive AF bacterial culture and an absolute PMN count ≥250 cells/mm^3^, and appears to easily identify patients who need empiric antibiotic coverage [51, 52] while waiting for final culture. Third-generation cephalosporins are used in patients with community-acquired SBP, and cure more than 80% of patients. However, in recent years, due to the widespread use of antibiotic prophylaxis and the increased frequency of hospitalization, the etiology of SBP is more related to multi-drug resistant bacteria [53, 54]. In light of this new and emergent scenario it is crucial to find an alternative treatment able to improve/restore the immune status of AF and limit bacterial overgrowth and SBP.

At present, the surgical approach with LT is considered the gold standard for treating end-stage liver diseases, even though the success of LT entails several challenges, namely the need to overcome the shortage of donor organs and the importance of proper recipient selection [55]. A regenerative medicine approach to liver disease has recently been considered a new tool for dealing with the current shortage of donor livers available for transplantation [56]. Some clinical trials have used mesenchymal stromal cells (MSCs) as cell therapy for liver disease treatment, based on their anti-inflammatory and immunomodulatory capabilities [57–59].

The purpose of this study was to use MSCs obtained from term human placenta (hA-MSCs), with the aim of assessing over time both the inflammatory and immunological state of the AF (obtained from cirrhotic patients) following treatment with hA-MSCs. We first demonstrated the capability of hA-MSCs to survive and proliferate in AF up to 1 week when grown in contact with AF. When compared with the growth under standard condition (with RPMI), the hA-MSC growth was affected by short-term (acute) exposure of both A(SN) and A(C). Even if hA-MSCs responded to AF first with an increase of cell death, maybe due to an acute damage- or stress-related events after 8 h and 24 h, they increased the proliferation rate in parallel with a decreased oxidative stress after 72 h, and recovery after 1 week. Because vascular endothelial growth factor (VEGF) regulates not only vasculogenesis and angiogenesis, but also cell proliferation and migration (Christopher R. Schlieve et al., 2016), our data correlated with the increase of VEGF-A production at the time points chosen. The hA-MSCs were able to respond to AF exposure, reducing apoptosis and necrosis after 1 week of exposure, suggesting a resistance to stress (revealed at 24 and 72 h), even when compared with cells grown in RPMI, in which the unchanged culture medium for a long time determined cell death and ROS production. These finding suggest a possible adaptation and survival of hA-MSCs in the AF environment. Moreover, the effects due to the AF exposure seemed not to induce any change in hA-MSC morphology or classic MSC marker expression.

The analyses of cytokines, growth factors, and leukocyte (specifically, macrophages, NK and T cells) profiles on post-paracentesis AF were also carried out after co-culture with hA-MSCs. Results showed that there was no statistically significant increase in EGF, IL-1β and IL-6 cytokines, while TNF-α release significantly increased starting from 72 h, and retained up to 1 week, which allows us to affirm that the presence of hA-MSCs can avoid the immune paralysis that often occurs in patients with advanced cirrhosis [60]. This was also concomitant with the high levels of T cells that were maintained by TNF-α, even in the presence of hA-MSCs. The co-culture with hA-MSCs determined a significant increase in IL-12p40 at 72 h compared to 24 h, and IL-4 (anti-inflammatory cytokine that stimulates M2 status), which significantly increased at 72 h compared to both 1 h and 24 H*. IL*-12 is a cytokine secreted by mononuclear phagocytes and dendritic cells, and is expressed following activation of toll-like receptors (TLRs) via microbial pathogen-associated molecular patterns (PAMP), mainly viruses and intracellular bacteria [61]. Its expression is also induced by the activation of phagocytes and dendritic cells by T-helper lymphocytes. In the latter case, this occurs thanks to the binding of CD40L of the T-helper lymphocyte with the corresponding receptor, CD40, expressed on the plasma membrane of the phagocyte or dendritic cell [61, 62]. After paracentesis, M1-like polarization was prevalent, surely due to the presence of an inflammatory state of the AF. Macrophages are highly plastic cells, which can respond to subtle changes in the tissue microenvironment by initiating several activation programs. The activation of macrophages occurs according to two main types of program: the classic inflammatory activation (M1), of which the activating stimuli are bacterial molecules (e.g., LPS) and inflammatory cytokines, and the alternative activation (or M2), stimuli activators of which are the anti-inflammatory cytokines (e.g., IL-4), immune complexes or glucocorticoids [32, 63]. The initial inflammatory response activates the M1 polarization of macrophages, which become able to eliminate the invading new microorganisms, and promote the inflammatory response, while during the inflammation resolution phase, in which the increase of IL-4 plays a determining role, the macrophages are repolarized in the M2 direction, losing their reactivity to inflammatory stimuli, and assuming the ability to eliminate damaged cells and tissues, and to promote angiogenesis and tissue repair [64]. The same analysis was carried out in the presence of LPS in order to mimic a condition of uncomplicated ascites infection. Even in the presence of LPS, hA-MSCs determined an increased M2-like expression of macrophages, which was significantly higher at 72 h (compared to 1 h and 24 h), while after 1 week of co-culture, both in the absence or presence of LPS, M2-like cells decreased compared to72 h. Though the presence of hA-MSCs, with or without LPS, caused an increase in M2-like macrophages at 72 h and at 1 week in both cases, the M1-like component increased in the presence of MSCs with LPS at 72 hours compared to 24 h, while after 1 week, M1-like macrophages were significantly higher than 24 h, demonstrating that hA-MSCs may not interfere with macrophages present in the AF of cirrhotic patients. This suggests that macrophages maintain the phagocytic activity to eliminate bacteria that translocate into AF in advanced cirrhosis, and the decrease of M2-like macrophages can indicate the resolution of the antimicrobial phase. Compared with cytokines present in AF soon after paracentesis, the presence of hA-MSCs determined a significant increase in IFN-γ and IL-2 at 72 h compared to 1 h and 24 h. This may explain the increase in the M1 component that still maintains the anti-microbial activity despite the significant shift towards an M2-like state. IL-2 stimulates the survival, proliferation, and differentiation of T cells activated by antigens by inducing the synthesis of IFN-γ. However, the IFN-γ is produced in response to stimulation of microbes by NK cells, but this production shares many characteristics with T cells. IL-2 also induces the proliferation of NK cell differentiation, and enhances their cytotoxic function [65].These were confirmed by the significant increase in NK after 72 h of co-culture with hA-MSCs compared to 24 h, followed by a subsequent reduction after 1 week. This suggests, therefore, that the presence of hA-MSCs is capable of creating an environment that favors the elimination of bacterial components through production of IFN-γ/IL-2, and an increase of M1-like macrophages and NK at 72 h. Starting at 72 h, however, a resolution of the inflammatory state was obtained due to the simultaneous increase of the anti-inflammatory M2 component up to 1 week, in which the M1 component instead appeared to decrease together with the M2-like macrophages and NK cells. Only T cells were maintained at higher levels due to higher release of TNF-α.

These findings suggest that hA-MSCs can be considered a new strategic cell therapy in liver cirrhosis complicated by refractory ascites, because they are able to restore the immune impairment in the ascites by modulating cytokine expression and cell response.

In particular, our work demonstrates, for the first time to the best of our knowledge, that cellular and cytokine profiles of ascites change toward an anti-inflammatory and anti-microbial cell phenotype in presence of hA-MSCs. The next important step will be to evaluate the effect of hA-MSCs in presence of ascites with bacterial infection, moving quickly into in vivo animal model testing.

## Supplementary Information

ESM 1 (DOCX 1689 kb)

## Data Availability

The datasets generated during and analysed during the current study are available from the corresponding author on reasonable request.

## Abbreviations

hA-MSCs: human amnion-derived mesenchymal stromal cells
CLD: chronic liver disease
LT: liver transplantation
SBP: spontaneous bacterial peritonitis
PH: portal hypertension
AF: ascitic fluid
BM-MSCs: bone marrow mesenchymal stromal cells
hAECs: human amnion-derived epithelial cells
PMN: polymorphonuclear leukocyte
A(SN): supernatant ascitic fluid
A(C): complete ascitic fluid
RLU: relative luminescence units
LPS: lipopolysaccharide
