# Supplementary material for: Human Amnion-Derived Mesenchymal Stromal Cells in Cirrhotic Patients with Refractory Ascites: A Possible Anti-Inflammatory Therapy for Preventing Spontaneous Bacterial Peritonitis
Source: Stem Cell Rev Rep. 2021 Jan 3;17(3):981–98. doi: 10.1007/s12015-020-10104-8 (PMC8166706; doi:10.1007/s12015-020-10104-8)
Supplement: Supplementary file 1 — (DOCX 1689 kb) [file 12015_2020_10104_MOESM1_ESM.docx]

**Supplementary Figure 1.**


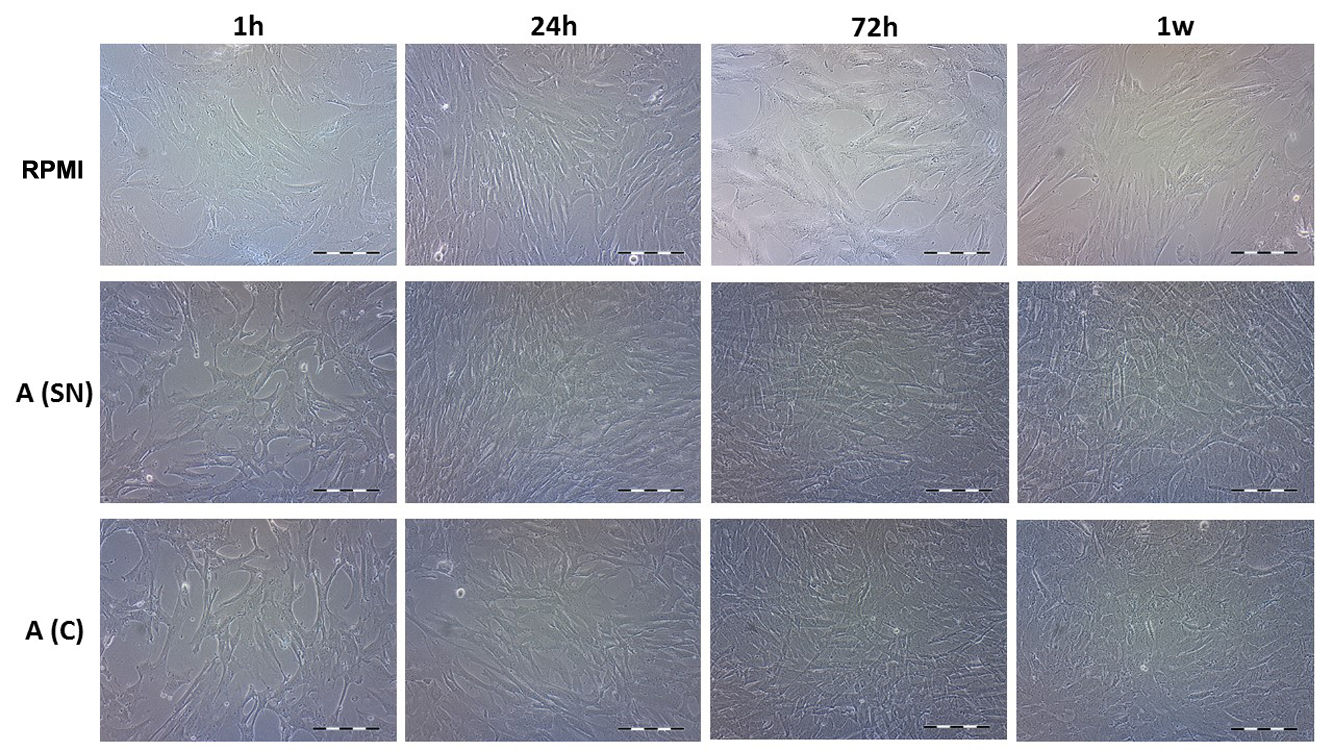


Panel of micrographs at phase contrast microscope showing hA-MSCs cultured in standard medium (RPMI), A(SN), and A(C) after 1 hour, 24 hours, 72 hours and 1 week, which maintained their morphological fibroblast-like shape during all the time point sets. Scale bar = 200 µm.
